# Correlation between extraocular muscle enlargement and thyroid autoantibodies in thyroid eye disease

**DOI:** 10.1007/s10384-024-01061-7

**Published:** 2024-04-13

**Authors:** Takahiro Koizumi, Takahiro Tanaka, Kazuki Umeda, Daisuke Komiyama, Hiroto Obata

**Affiliations:** Department of Ophthalmology, Saitama Medical Center, 1981 Kamoda, Kawagoe, Saitama 350-8550 Japan

**Keywords:** Extraocular muscle enlargement, Thyroid autoantibody, Thyroid eye disease, TRAb, TSAb

## Abstract

**Purpose:**

This study aimed to investigate the factors affecting extraocular muscle enlargement in thyroid eye disease (TED).

**Study design:**

Retrospective study.

**Methods:**

The thyroid-stimulating hormone (TSH) receptor antibody (TRAb), thyroid-stimulating antibody (TSAb), antithyroid peroxidase antibody (ATPO), and antithyroglobulin antibody (ATG) levels in patients diagnosed with TED who underwent orbital magnetic resonance imaging were assessed. The control group comprised the contralateral eye of patients who underwent orbital magnetic resonance imaging (MRI) for unilateral eyelid tumors or orbital disease. The thickness of the bilateral rectus muscles and superior oblique muscles was measured on orbital MRI. Muscle enlargement was classified as unilateral/bilateral and symmetric/asymmetric. The effects of age, sex, smoking history, TSH, thyroid hormone, and thyroid autoantibodies on the muscle thickness and number of enlarged muscles were assessed by use of simple and multiple regression analyses.

**Results:**

The TED and control groups comprised 41 and 44 cases, respectively. The positivity rate of TSAb in patients with TED was 92.7% higher than that of the other autoantibodies. Muscle enlargement was observed in 29 of the 41 cases (70.7%). Older age and higher TSAb levels were identified as significant factors affecting the total muscle thickness and number of enlarged muscles. Bilateral muscle enlargement and asymmetrical muscle enlargement were observed in 17 (58.6%) and 23 (79.3%) of the 29 cases, respectively. The TSAb levels and age had no significant effect on the type of muscle enlargement.

**Conclusions:**

TSAb showed significant associations with extraocular muscle enlargement. Measurement of TSAb, rather than of TRAb, may be more useful for diagnosing extraocular muscle enlargement in patients with TED.

## Introduction

Thyroid eye disease (TED) occurs in 25–50% of cases of hyperthyroidism (Graves disease) and approximately 2% of cases of hypothyroidism (Hashimoto disease). TED is an autoimmune inflammatory disease characterized by the presence of inflammatory changes in the extraocular muscles, orbital adipose tissue, eyelids, and lacrimal glands [1].

Thyroid-stimulating hormone receptor antibody (TRAb) and thyroid-stimulating antibody (TSAb) [2] are known biomarkers for TED. Several studies have reported the effectiveness of TRAb and TSAb. However, which of these 2 biomarkers could be superior remains unclear [2–7]. A small number of cases of TED are positive for antithyroid peroxidase antibody (ATPO) and antithyroglobulin antibody (ATG), which are associated with Hashimoto disease. However, only one report has investigated the association of ATPO and ATG with TED [8].

Eye movement disorders and diplopia due to enlargement of the extraocular muscles have adverse effects on the activities of daily living in patients with TED. Significant muscle enlargement may lead to loss of visual function owing to compression of the optic nerve. The clinical activity score (CAS) is used to evaluate the activity of TED, whereas the NOSPECS classification (no signs or symptoms, only signs, soft tissue involvement, proptosis, extraocular muscle involvement, corneal involvement, and sight loss) by the American Thyroid Association is used to evaluate the severity of TED. Previous studies have investigated the associations of TRAb/TSAb with CAS and TRAb/TSAb with diplopia according to the NOSPECS classification [3–7]. However, few studies have examined the association between TRAb/TSAb and muscle enlargement in detail [8–10]. Moreover, no study has examined the association between muscle enlargement and these 4 thyroid autoantibodies (TRAb, TSAb, ATPO, and ATG).

The symptoms of TED vary according to age. Orbital fat expansion is the most common finding in patients aged younger than 40 years, whereas muscle enlargement is the most common finding in patients aged 60 years or older [1]. Most cases of TED are bilateral. However, unilateral cases and cases of laterality have also been reported [1, 11–14]. The extraocular muscles comprise 6 pairs of muscles. Muscle enlargement may occur only in 1 eye, or the number of enlarged muscles may differ between the right and left eyes. The pathogenesis of unilateral and asymmetric cases and the mechanism of TED remain unknown. Therefore, in this study, we investigated the effect of age, sex, smoking history, thyroid-stimulating hormone (TSH), thyroid gland hormone, TRAb, TSAb, ATPO, and ATG on the thickness of 5 pairs of extraocular muscles and the number of enlarged muscles. In addition, we investigated the effect of the antibody levels on bilateral/unilateral and symmetric/asymmetric muscle enlargement.

## Patients and methods

### Patients

This study was approved by the ethics committee of the Saitama Medical Center (approval number: SOU2021-015) and adhered to the tenets of the Declaration of Helsinki. The patients were provided with an opportunity to opt out of the study on the hospital’s website.

The medical records of consecutive patients who were diagnosed with TED at the Department of Ophthalmology, Saitama Medical Center, between April 1, 2017 and March 31, 2023 were retrospectively reviewed. The patients who had undergone measurement of TSH, thyroid hormone, TRAb (3rd generation), TSAb, ATPO, and ATG levels, along with orbital magnetic resonance imaging (MRI), were included in this study. Eleven patients with no recorded measurements for 1 of the 4 antibodies, 5 patients with a history of steroid and immunosuppressant drugs use, 3 patients who had not undergone MRI examinations, 2 patients with blurred MR images, and 2 patients who had received treatment for TED were excluded.

The MR images of the contralateral eye in patients who had undergone MRI examinations for unilateral eyelid or orbital tumors were used as the controls. Measurements were obtained from a total of 10 extraocular muscles: the bilateral superior rectus muscles (SR), inferior rectus muscles (IR), lateral rectus muscles (LR), medial rectus muscles (MR), and superior oblique muscles (SO).

MRI examinations were performed using MAGNETOM Verio or Skyra Fit (Siemens). The section thickness was 3 mm, and simple T1-weighted images were used for evaluation.

### Muscle thickness and muscle enlargement

**Fig. 1:**
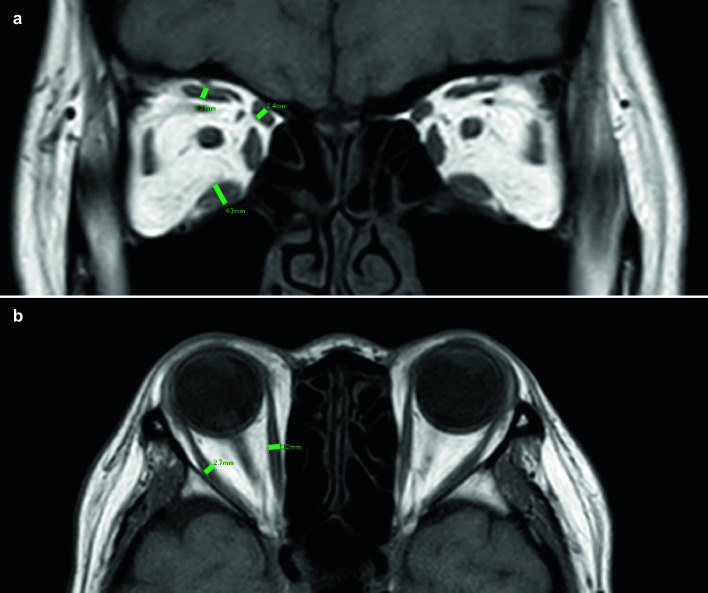
Measurement of extraocular muscle thickness. **a** The thicknesses of the SR, IR, and SO were measured on coronal sections of orbital MRI as the short axis of the thickest part in each muscle. **b** The thicknesses of the MR and the LR thickness were measured on horizontal sections of orbital MRI. *SR* superior rectus muscle, *IR* inferior rectus muscle, *LR* lateral rectus muscle, *MR* medial rectus muscle, *SO* superior oblique muscle, *MRI* magnetic resonance imaging

Muscle thickness was measured using the I-PACS VR system (Konica Minolta). The thicknesses of the SR, IR, and SO were measured on the coronal sections, whereas the thicknesses of the MR and LR were measured on the horizontal sections. The length of a line perpendicular to the long axis was considered the thickness of the muscle in the image with the maximum section thickness (Fig. 1a, b). Three ophthalmologists (T.K., K.U., and D.K.) performed the measurements independently; the average of the results was considered the muscle thickness. The mean and standard deviation (SD) of the thickness of each muscle in the control group was calculated. Muscle enlargement was defined as a thickness greater than mean + 2 SD of the muscle thickness of the control group [15]. The number of enlarged muscles in each case and the average number of enlarged muscles in all the cases were determined.

### Association of the total muscle thickness and the number of enlarged muscles with each factor

Simple and multiple regression analyses were performed using the total muscle thickness and number of enlarged muscles as objective variables and age, sex, smoking history, TSH, thyroid hormone, and each autoantibody level as explanatory variables to investigate the association of the total muscle thickness and the number of enlarged muscles with each factor.

### Association of bilateral/unilateral and symmetric/asymmetric with age and TSAb

**Fig. 2:**
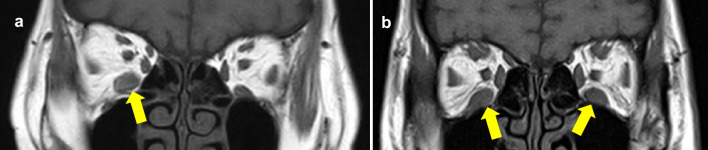
Representative bilateral/unilateral and symmetric/asymmetric cases of extraocular muscle enlargement. **a** This case showed only IR enlargement in the right eye and was classified as a unilateral and asymmetric case. **b** This case showed IR enlargement in both eyes and was classified as a bilateral and symmetric case. *IR* inferior rectus muscle

In this study, cases of muscle enlargement in both eyes were defined as bilateral cases. Cases of muscle enlargement in only 1 eye were defined as unilateral cases. Cases of enlargement of the same muscle in both eyes were defined as symmetric cases. Cases of muscle enlargement, in only 1 eye or enlargement on both sides but with the involvement of different muscles were defined as asymmetric cases (Fig. 2). The associations of bilateral/unilateral and symmetric/asymmetric muscle enlargement with age and TSAb were evaluated.

### Statistical analysis

EZR was used to perform all statistical analyses. EZR is a statistical software program that extends the functions of R and R Commander [16]. Factors with *P* < 0.1 from the simple regression analysis were fitted into the multiple regression analysis, and independent variables with *P* < 0.05 were selected using the step-down method. The Mann–Whitney U test was used to compare the groups, and *P* values < 0.05 were considered significant.

## Results

### Patients’ characteristics

**Fig. 3:**
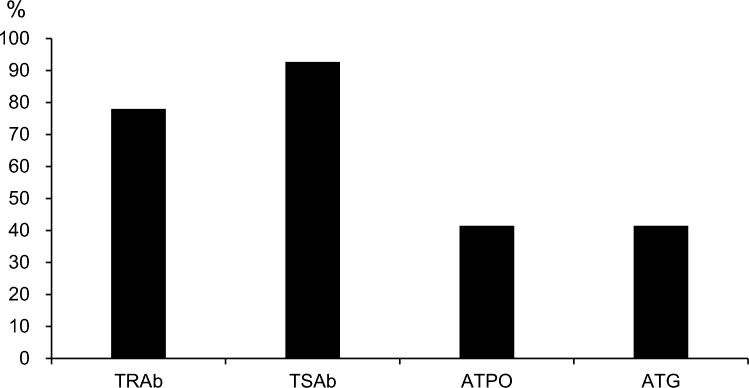
Positivity rates for each thyroid autoantibody. The positivity rates for each antibody in 41 patients with TED were as follows: TRAb, 78.0%; TSAb, 92.7%; ATPO, 41.5%; and ATG, 41.5%. The positivity rate of TSAb was higher than that of TRAb. *TRAb* thyroid-stimulating hormone receptor antibody, *TSAb* thyroid-stimulating antibody, *ATPO* antithyroid peroxidase antibody, *ATG*, antithyroglobulin antibody

The TED group included 41 patients (9 men and 32 women; mean age, 55.2 ± 13.9 years; range 27–77 years) (Table 1), whereas the control group included 44 patients (24 men and 20 women; mean age, 61.4 ± 14.8 years; range 29–88 years) (Table 2). Among the 26 patients in the TED group who had received treatment for thyroid dysfunction, 24 had received oral medications, two had received radioisotope therapy, and one had undergone surgery. Fifteen patients had no history of receiving treatment and 7 patients were smokers. The mean (SD) values for thyroid hormone, TSH, and autoantibodies were as follows: free T3, 4.38 (3.15) pg/mL; free T4, 1.67 (1.39) ng/dL; TSH, 4.83 (16.38) μIU/mL; TRAb, 102.3 (478.8) IU/L; TSAb, 1317.7% (1407.8); ATPO, 237.3 (657.5) IU/mL; and ATG, 130.96 (231.9) IU/mL. Furthermore, 25 patients had lid retraction, 30 patients had eyelid swelling, 21 patients had ocular movement disorder, 18 patients had proptosis, and 1 patient had optic neuropathy. The positivity rates for each antibody in the TED group were as follows: TRAb, 78.0%; TSAb, 92.7%; ATPO, 41.5%; and ATG, 41.5% (Fig. 3).

**Table 1:** Characteristics of thyroid eye disease group

Number of cases	41
Age, years	55.2 (13.9): 22–77^a^
Sex, male/female	9/32
Treatment history (at visited)	
Oral medication/radioiodine therapy/surgery/none	24/2/1/15
Smoking (at visited)	7
Laboratory data (normal range)	
Free T3 (2.3–4.0), pg/mL	4.38 (3.15): 0.87–15.2^a^
Free T4(1.0–1.8), ng/dL	1.67 (1.39): 0.17–8.47^a^
TSH (0.50–5.00), μIU/mL	4.83 (16.38): 0.01–100^a^
TRAb (0–2.0), IU/L	102.3 (478.8): 0.3–3110.0^a^
TSAb (0–120) (%)	1317.7 (1407.8): 103.0–4747.0^a^
ATPO (0–15), IU/mL	237.3 (657.5): 2.1–3533.4^a^
ATG (0–27), IU/mL	131 (231.9): 10.0–1053.0^a^
Clinical findings (no. of cases)	
Eyelid retraction	25
Eyelid swelling	30
Ocular movement disorder	21
Proptosis	18
Optic neuropathy	1
Thickness of extraocular muscles (mm) (Right eye/left eye)	
SR	2.86 (0.92): 1.70–6.10^a^/2.84 (1.14): 1.43–6.80^a^
IR	4.71 (1.29): 2.90–8.87^a^/4.59 (0.93): 2.77–6.77^a^
LR	3.75 (0.62): 2.50–5.30^a^/3.79 (0.74): 2.63–6.50^a^
MR	3.93 (0.98): 2.87–6.60^a^/4.07 (1.34): 2.67–8.93^a^
SO	2.54 (0.45): 1.77–3.87^a^/2.61 (0.62): 1.83–4.60^a^

*TRAb* thyroid-stimulating hormone receptor antibody, *TSAb* thyroid-stimulating antibody, *ATPO* antithyroid peroxidase antibody, *ATG* antithyroglobulin antibody, *SR* superior rectus muscle, *IR* inferior rectus muscle, *LR* lateral rectus muscle, *MR* medial rectus muscle, *SO* superior oblique muscle

^a^Mean (standard deviation): range

**Table 2.** Characteristics of the control group

Number of cases	44
Sex, male/female	24/20
Age, years	61.4 (14.8): 29–88^a^
Thickness of extraocular muscles, mm	
SR	2.36 (0.33): 1.53–3.17^a^
IR	3.94 (0.54): 2.80–5.07^a^
LR	3.58 (0.50): 2.60–4.83^a^
MR	3.60 (0.34): 3.0–4.30^a^
SO	2.44 (0.27): 1.93–3.20^a^

*SR* superior rectus muscle, *IR* inferior rectus muscle, *LR* lateral rectus muscle, *MR* medial rectus muscle, *SO* superior oblique muscle

^a^Mean (standard deviation): range

### Muscle thickness

The average (SD) thickness of each muscle in the TED group was as follows: In the right eye, it was SR, 2.86 (0.92) mm; IR, 4.71 (1.29) mm; LR, 3.75 (0.62) mm; MR, 4.0 (0.86) mm; and SO, 2.54 (0.45) mm. In the left eye, it was SR, 2.84 (1.14) mm; IR, 4.59 (0.93) mm; LR, 3.79 (0.93) mm; MR, 4.24 (1.15) mm; and SO, 2.61 (0.62) mm (Table 1). The average (SD) thickness of each muscle in the control group was SR, 2.36 (0.33) mm; IR, 3.94 (0.54) mm; LR, 3.58 (0.54) mm; MR, 3.66 (0.41) mm; and SO, 2.44 (0.27) mm (Table 2).

### Muscle enlargement

**Fig. 4:**
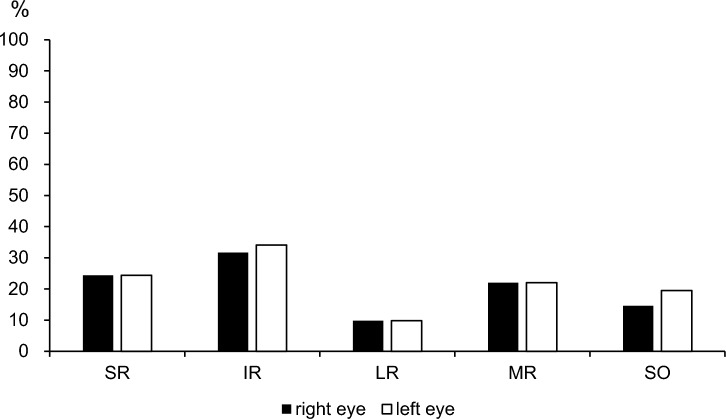
Incidence of enlarged extraocular muscles in each eye. The IR was the most frequently enlarged muscle in patients with TED

Muscle enlargement was observed in 29 of the 41 patients with TED (70.7%). In the right eye, enlargement was observed in the SR (10 eyes, 24.4%), IR (13 eyes, 31.7%), LR (4 eyes, 9.8%), MR (9 eyes, 22.0%), and SO (6 eyes, 14.6%). In the left eye, enlargement was observed in the SR (10 eyes, 24.4%), IR (14 eyes, 34.1%), LR (4 eyes, 9.8%), MR (9 eyes, 22.0%), and SO (8 eyes, 19.5%) (Fig. 4). The IR was the most frequently affected muscle.

The numbers of enlarged muscles were as follows: 12 patients, 0 enlarged muscles (29.3%); 9 patients, 1 enlarged muscle (22.0%); 8 patients, 2 enlarged muscles (19.5%); 3 patients, 3 enlarged muscles (7.3%); 3 patients, 4 enlarged muscles (7.3%); 1 patient, 5 enlarged muscles (2.4%); 2 patients, 6 enlarged muscles (4.9%); 1 patient, 7 enlarged muscles (2.4%); 1 patient, 8 enlarged muscles (2.4%); and 1 patient, 9 enlarged muscles (2.4%). Enlargement of all 10 muscles was not observed in any patient. The average number of enlarged muscles was 2.1 muscles.

### Association of the total muscle thickness and the number of enlarged muscles with each factor

A simple regression analysis was performed to determine the association between the total muscle thickness and each factor. Older age, lower free-T4, and higher TSAb (Table 3) were identified as significant factors. Multiple regression analysis revealed that older age and higher TSAb were the significant factors affecting the total muscle thickness (Table 4).

**Table 3:** Factors affecting the total thickness of the extraocular muscles (simple regression analysis)

	Coefficients	SE	t value	*P* value	95% CI
Age	0.165	0.061	2.693	0.010*	0.041 to 0.289
Sex	3.274	2.185	1.498	0.142	− 1.146 to 7.694
Smoking	0.534	2.471	0.216	0.830	− 4.463 to 5.531
Free T3	− 0.325	0.291	− 1.117	0.271	− 0.912 to 0.263
Free T4	− 1.537	0.622	− 2.473	0.018*	− 2.794 to − 0.280
TSH	0.033	0.057	0.590	0.558	− 0.081 to 0.148
TRAb	0.002	0.002	0.899	0.374	− 0.002 to 0.006
TSAb	0.002	0.001	3.696	0.001*	0.0009 to 0.003
ATPO	− 0.001	0.001	− 0.451	0.655	− 0.003 to 0.002
ATG	0.007	0.004	1.720	0.093*	− 0.001 to 0.014

*SE* standard error, *Free-T4* free thyroxine, *Free-T3* free triiodothyronine, *TSH* thyroid-stimulating hormone, *TRAb* thyroid-stimulating hormone receptor antibody, *TSAb* thyroid-stimulating antibody, *ATPO* antithyroid peroxidase antibody, *ATG* antithyroglobulin antibody

*Significant *P* value (*P* < 0.1 is considered significant)

**Table 4:** Factors affecting the total thickness of the extraocular muscles (multiple regression analysis)

	Coefficients	SE	t value	*P* value	95% CI
Age	0.153	0.053	2.897	0.006*	0.046 to 0.260
TSAb	0.002	0.001	3.849	< 0.001*	0.001 to 0.003

*SE* standard error, *TSAb* thyroid-stimulating antibody

*Significant *P* value (*P* < 0.05 is considered significant)

A simple regression analysis was performed to determine the association between the number of enlarged muscles and each factor. Older age, lower free-T4, and higher TSAb were identified as the factors affecting the number of enlarged muscles (Table 5). Multiple regression analysis revealed that age and TSAb were the significant factors affecting the number of enlarged muscles (Table 6).

**Table 5:** Factors affecting the number of enlarged extraocular muscles (simple regression analysis)

	Coefficients	SE	t value	*P* value	95% CI
Age	0.073	0.024	3.009	0.005*	0.024 to 0.121
Sex	1.010	0.886	1.140	0.261	− 0.782 to 2.803
Smoking	0.542	0.987	0.549	0.586	− 1.455 to 2.539
Free T3	− 0.138	0.116	− 1.188	0.242	− 0.373 to 0.097
Free T4	− 0.528	0.254	− 2.074	0.045*	− 1.042 to − 0.013
TSH	0.027	0.022	1.211	0.233	− 0.018 to 0.072
TRAb	0.001	0.001	1.363	0.181	− 0.001 to 0.003
TSAb	0.001	0.000	3.573	0.001*	0.0003 to 0.001
ATPO	0.000	0.001	− 0.573	0.570	− 0.001 to 0.001
ATG	0.002	0.002	1.113	0.272	− 0.001 to 0.005

*SE* standard error, *Free-T4* free thyroxine, *Free-T3* free triiodothyronine, *TSH* thyroid-stimulating hormone, *TRAb* thyroid-stimulating hormone receptor antibody, *TSAb* thyroid-stimulating antibody, *ATPO* antithyroid peroxidase antibody, *ATG* antithyroglobulin antibody

*Significant *P* value (*P* < 0.1 is considered significant)

**Table 6:** Factors affecting the number of enlarged extraocular muscles (multiple regression analysis)

	Coefficients	SE	t value	*P* value	95% CI
Age	0.068	0.021	3.251	0.002*	0.026 to 0.110
TSAb	0.001	0.000	3.785	< 0.001*	0.0004 to 0.0012

*SE* standard error, *TSAb* thyroid-stimulating antibody

*Significant *P* value (*P* < 0.05 is considered significant)

### Associations of bilateral/unilateral and symmetrical/asymmetrical muscle enlargement with age and TSAb

Among the 29 patients with muscle enlargement, 17 patients (58.6%) had bilateral muscle enlargement, and 12 patients (41.4%) had unilateral muscle enlargement (Table 7). Six patients (20.7%) had symmetric muscle enlargement, and 23 patients (79.3%) had asymmetric muscle enlargement; the number of asymmetric cases was larger. The association of age and TSAb with bilateral/unilateral and symmetrical/asymmetrical muscle enlargement was evaluated. However, age and TSAb were found to have no effect on bilateral/unilateral and symmetrical/asymmetrical muscle enlargement.

**Table 7:** Association of age and TSAb with bilateral/unilateral and symmetric/asymmetric cases with enlarged extraocular muscles

	Bilateral/unilateral	Symmetry/asymmetry
No. of cases	17/12	6/23
Age, years	61.1 (11.2): 39–77^a^/56.7 (13.9): 38–73^a^	61.2 (10.2): 39–68^a^/58.7 (12.2): 38–77^a^
*P* value	0.66	0.89
Sex		
Male	4/1	2/3
Female	13/11	4/20
TSAb	1759.1 (1471.3): 106.0–4747.0^a^/1116.1 (1305.1): 103.0–4540.0^a^	2370.8 (1533.2): 186.0–4747.0^a^/1264.1 (490.6): 103.0–4540.0^a^
*P* value	0.18	0.22

*TSAb* thyroid-stimulating antibody

Mann–Whitney U test

^a^Mean (standard deviation): range

## Discussion

This study was conducted to identify the factors associated with extraocular muscle enlargement in patients with TED. To this end, the effects of age, sex, smoking history, TSH, thyroid hormone, TRAb, TSAb, ATPO, and ATG on the muscle thicknesses of 10 extraocular muscles and the number of enlarged muscles was investigated by use of MRI. Age and TSAb were found to be significantly associated with the total extraocular muscle thickness and number of enlarged muscles, respectively.

Diplopia and compressive optic neuropathy are significant symptoms of TED that occur because of extraocular muscle enlargement. TED is thought to be an autoimmune disorder in which the autoantigen acts on the TSH receptors of CD34-positive fibroblasts in the orbital tissue [17–20]. Activated fibroblasts and lymphocytes infiltrating the orbit produce cytokines. In addition, activated fibroblasts produce glycosaminoglycans, such as hyaluronic acid, and are presumed to cause inflammatory hypertrophy and fibrosis in extraocular muscles and retrobulbar tissues. However, the mechanism of extraocular enlargement remains unclear owing to the difficulty in obtaining extraocular muscle tissue, treatment-induced alterations in the tissue, and the lack of a useful animal model of TED. A previous study on extraocular muscles revealed infiltration of lymphocytes and plasma cells and deposition of glycosaminoglycans [21]. The lymphocytes were mainly CD4-positive T lymphocytes, which have been reported to produce various cytokines, such as interleukin-4 [22]. Therefore, the present study focused on the factors associated with extraocular muscle enlargement in patients with TED.

Various methods are available for evaluating extraocular muscle enlargement by use of MRI and CT images, such as measurements of muscle volume, cross-sectional area, and muscle thickness [15, 23–30]. The muscle thickness was measured and examined in the present study [15, 29, 30]. The thickness of the extraocular muscles varies from person to person, making it difficult to identify pathologic enlargement of the muscles. Therefore, the mean+2 SD thickness of each muscle in the control group was used as a cutoff point to determine muscle enlargement [15]. In the present study, the IR was the most frequently affected muscle, followed by the SR and MR, which were affected almost equally. The IR and MR are the most commonly affected muscles in TED. However, reports have been published of the SR being affected more frequently than the MR [23–27].

Previous studies have assessed the presence or absence of diplopia in patients by using TRAb, TSAb, and the CAS and NOSPECS classification [3–7]. However, these studies mainly assessed the association between the clinical scores and autoantibodies. Moreover, these studies did not determine the number of extraocular muscles or the muscle enlargement quantitatively by measuring the thickness. Few reports have assessed the association between autoantibodies and muscle enlargement [8–10]. Kvetny and colleagues [8] analyzed the association of MR volume measured by using MRI with TRAb, ATPO, TSH, and thyroid hormone. A correlation was found between the MR volume and TRAb, but not ATPO, TSH, and thyroid hormone. The association of TSAb with MR volume was not investigated. Hiromatsu and colleagues [9] investigated the association between TSAb measured using CHO cells and the 4 rectus muscles using MRI and reported no correlation with the area and number of enlarged muscles. However, an association was observed with the signal intensity of the MRI. A detailed description of the method was not provided. Therefore, whether the area of the enlarged muscle was the sum of the 4 muscles remains unclear; the muscle targeted by the signal intensity is not known. In addition, the association between TRAb and muscle enlargement was not investigated. Choi and colleagues [10] categorized 65 cases of TED with extraocular muscle restriction due to muscle enlargement into improved (32 cases) and not-improved (33 cases) groups. Examination of the factors that affect eye movement restriction revealed that a significant difference was only observed in the TSAb level. In other words, the TSAb level before treatment was lower in the improvement group than that in the non-improvement group. Thus, they concluded that TSAb was superior to TRAb for evaluating restrictive myopathy. In the present study, the thicknesses of a total of 10 extraocular muscles (4 rectus muscles and superior oblique muscles on both sides) were measured by use of MRI images. The association between 2 indices of extraocular myomegaly, the total muscle thickness and number of enlarged muscles, and 4 autoantibodies and thyroid hormones were investigated. To the best of our knowledge, no such a study has been conducted previously. The findings of the present study show that among the 4 autoantibodies, only TSAb was significantly associated with extraocular muscle enlargement.

The symptoms of TED tended to differ according to age, and muscle enlargement is said to be common in patients aged 60 years or older [1]. This study evaluated the association of the total muscle thickness and number of enlarged muscles with age, sex, smoking history, TSH, thyroid hormone, and autoantibody levels and found significant associations with TSAb and age. However, no correlation was observed between age and TSAb. The causes of the variations in the TED phenotype according to age are unclear. Kazim and colleagues [31] reported that the fibroblasts of young patients with TED are prone to adipogenic differentiation. However, the adipogenic potential decreases with aging, and nonadipogenic fibroblasts are predominant in older patients with TED, which promotes inflammation and fibrosis.

Although most cases of TED are bilateral, unilateral cases and cases of laterality have been reported [1, 11–14], leading to a delay in TED diagnosis. The pathophysiology of unilateral and asymmetric cases is unknown. However, some studies have shown that the activity and severity of asymmetric or unilateral cases are higher than those of symmetric or bilateral cases [11–13], whereas some reports have shown no such differences [14]. In the present study, extraocular muscle enlargement was more common in bilateral cases than in unilateral cases. Similarly, extraocular muscle enlargement was more common in asymmetric cases than in symmetric cases. This study investigated the association of age and TSAb with bilateral/unilateral and symmetric/asymmetric muscle enlargement. However, no significant association was found. Unilateral cases may progress to bilateral involvement [32]. Therefore, further research must be conducted on the pathogenesis of unilateral and asymmetric TED.

Several studies have attempted to identify which of the 2 thyroid antibodies, TSAb and TRAb, is the superior biomarker for TED. However, the results have been inconsistent [2]. TRAb levels are measured by use of a competition immunoassay, which can detect its ability to compete with a labeled ligand (a monoclonal antibody to TSH receptor) for binding with the TSH receptor. In contrast, TSAb levels are measured by use of a bioassay, which can measure the production of cAMP using porcine thyroid cells with TSH receptors. The results of the TRAb analysis can be obtained within the same day at a hospital facility. In contrast, 5 to 7 days are required to obtain the results of the TSAb test because it is an outsourced test. Extraocular muscle enlargement has been observed on the MR images of patients with Graves disease who had no clinical ocular signs and symptoms may present [33]. TED with normal thyroid hormone levels (euthyroid Graves disease) is a rare entity. However, its incidence has been reported [34]. Thus, measuring the autoantibody levels, to ensure that the diagnosis of TED is not overlooked, is important. Although internists first measure TRAb, for which results are readily available, the findings of this study suggest that the primary measurement of TSAb is important for early detection and treatment of extraocular muscle enlargement in patients with TED.

This study has some limitations. First, it was a retrospective study with a small sample size. Second, this study did not consider the history of treatment for thyroid dysfunction. Owing to the small sample size, examining the effect of the presence or absence of treatment history for thyroid dysfunction separately was not possible. Third, this study evaluated extraocular muscle enlargement by use of MRI. The volume, cross- sectional area, and signal intensity, rather than the thickness, should have been measured to determine the activity to further evaluate the pathophysiology of extraocular muscle enlargement. However, measuring the volume and signal intensity of the images taken in this retrospective study was technically difficult. Further prospective studies with larger sample sizes must be conducted in the future.

In conclusion, the factors associated with extraocular muscle enlargement in patients with TED were older age and higher TSAb levels. Measurement of TSAb, rather than of TRAb, may be more suitable for diagnosing muscle enlargement in patients with TED.
